# Life expectancy estimations and determinants of return to work among cancer survivors over a 7-year period

**DOI:** 10.1038/s41598-021-92306-9

**Published:** 2021-06-18

**Authors:** Wei-Liang Chen, Yuan-Yuei Chen, Wei-Te Wu, Ching-Liang Ho, Chung-Ching Wang

**Affiliations:** 1 Division of Family Medicine, Department of Family and Community Medicine, Tri-Service General Hospital, and School of Medicine, National Defense Medical Center, Taipei, Taiwan, Republic of China; 2 Division of Occupational Medicine, Department of Family Medicine and Community Medicine, Tri-Service General Hospital, National Defense Medical Center, Number 325, Section 2, Chang-gong Rd, Nei-Hu District, 114, Taipei, Taiwan, Republic of China; 3 Division of Geriatric Medicine, Department of Family and Community Medicine, Tri-Service General Hospital, and School of Medicine, National Defense Medical Center, Taipei, Taiwan, Republic of China; 4 Department of Pathology, Tri-Service General Hospital Songshan Branch, and School of Medicine, National Defense Medical Center, Taipei, Taiwan, Republic of China; 5 Department of Pathology, Tri-Service General Hospital, and School of Medicine, National Defense Medical Center, Taipei, Taiwan, Republic of China; 6 National Institute of Environmental Health Sciences, National Health Research Institutes, Miaoli, Taiwan, Republic of China; 7 Division of Hematology/Oncology, Department of Medicine, Tri-Service General Hospital, and School of Medicine, National Defense Medical Center, Taipei, Taiwan, Republic of China; 8 Department of Biochemistry, National Defense Medical Center, Taipei, Taiwan, Republic of China

**Keywords:** Cancer, Health occupations

## Abstract

Due to advances in medical science and technology, the number of cancer survivors continues to increase. The workplace needs and employment difficulties cancer survivors face after treatment need to be addressed to protect these individuals’ right to work and to maintain the overall labor force of the country. We conducted a retrospective cohort study with a follow-up period from 2004 to 2010. All data analyzed in the study were obtained from the Labor Insurance Database, the Taiwan Cancer Registry of the Ministry of Health and Welfare, and the National Health Insurance Research Database. The relationships between risk factors and the presence of returning to work were analyzed by a Cox proportional hazard model. The survival rates of patients with different cancer stages were evaluated using Kaplan–Meier survival analysis. Among the employees with an initial diagnosis of cancer, 70.4% remained employed through 1 year after the diagnosis, accounting for 83.4% of all cancer survivors; only 51.1% remained employed through 5 years after the diagnosis, accounting for 78.7% of all cancer survivors, a notable decrease. Age, gender, salary, treatment method, company size, and cancer stage were the factors that affected whether employees could return to work or not. The long-term survival of people diagnosed with cancer depends on their chances of returning to work. Strengthening existing return-to-work policies and assisting cancer survivors with returning to work after the treatment should be priorities for protecting these individuals’ right to work and for maintaining the overall labor force.

## Introduction

The occupational safety and health of employees is the foundation of social stability. The protection of employee’s lives and improvements of their physical and mental health are critical for the stable development of society. With rapid industrial development, Taiwan’s employed population has increased for many years. According to the Taiwan Occupational Safety and Health Institute’s analysis of the causes of death between 2003 and 2008, malignant tumors were the leading cause of death among employees in Taiwan, with a standardized death rate of 47.0 per 100,000 people^1^. In Taiwan, pulmonary cancer has the highest mortality rate, followed by hepatic cancer, colorectal cancer, female breast cancer, gastric cancer, oral cancer, prostate cancer, cervical cancer, esophageal cancer, and pancreatic cancer. Among insured employees in Taiwan, liver cancer has the highest mortality rate, followed by pulmonary cancer, oral cancer, colorectal cancer, female breast cancer, esophageal cancer, gastric cancer, nasopharyngeal carcinoma, pancreatic cancer, and leukemia.

Because the incidence and prevalence of cancer has persistently increased in the past few years, along with the cure rates, cancer survivors are becoming increasingly more common in the workplace^2^. In addition to career interruptions and substantial medical expenses, a cancer diagnosis alone is a source of stress. These are all important factors that impact health-related quality of life. Most cancers affect patients at physical, mental and spiritual levels and even affect their functional and occupational abilities.

According to studies conducted in other countries, most employees choose to continue working during cancer treatment^3^. However, their employment was still interrupted by the treatment and its side effects, impacting their work efficiency. Cancer survivors after treatment have physiological, psychological, and social adaptation issues and are unable to immediately resume a full workload or work abilities similar to those before contacting the disease. According to a systemic review that included studies from Europe and Asia, the overall rate of returning to work estimated at was 72%^4^. Another cross-sectional study showed that 74% of breast cancer survivors expressed a desire to return work, but only 54% of these breast cancer survivors did so^5^. Cancer survivors have a high unemployment risk and may have considerable physical, psychological and social problems, such as fatigue, pain, cognitive decline, anxiety and depression, which may be temporary but may also persist and become chronic^6^. Current studies have noted that multiple comorbidities, poor health conditions, treatment complications, persistent pain, and depressive mood are important risk factors for unemployment^7^. After cancer treatment, many patients will undergo changes in work status, have taken prolonged sick leaves, and experience unemployment and various other issues, causing their chosen careers to end^8^. Therefore, evaluation and re-employment services for cancer survivors are necessary for the transition of these individuals from the disease state to the workplace.

Work disability places a great burden on individuals, the economy and public health. Therefore, successfully predicting and preventing work disability is an important research subject. Job participation can be regarded as an affirmation of self-worth, identity and social roles^9^. Returning to work or re-employment is very important for cancer survivors, their families and society. Most cancer survivors regard returning to work as a symbol of full recovery and regaining a normal life. Returning to work can also help maintain family income, self-esteem, sense of meaning, and health^10^. If unable to return to work, patients may be deprived of personal social contact and well-being^11^. Mehnert et al. summarized several studies and indicated that approximately 63.5% of cancer survivors will try to return to work after completing treatment; 6 months after diagnosis, approximately 40% of cancer survivors returned to work or kept working while receiving treatment (between 24 and 72%); 12 months after diagnosis, approximately 62% of cancer survivors returned to work or continued to work during treatment (between 50 and 81%); 18 months after diagnosis, approximately 73% of cancer survivors returned to the work or continued to work during treatment (between 64 and 82%); and 24 months after diagnosis, approximately 89% of cancer survivors returned to work or continued to work during treatment (between 84 and 94%)^12^. From the above studies, we found that due to different cancer characteristics and treatment guidelines, a certain proportion of people cannot return to their previous work; therefore, identifying risk factors, identifying high-risk groups, educating employers and employees, and applying existing return-to-work policies to assist cancer survivors are imminently needed.

Currently, there is still a lack of large-scale long-term follow-up studies on the return to work of cancer survivors in Taiwan. Therefore, the purpose of this study was to establish a long-term cohort database of cancer survivors based on the National Health Insurance Research Database and to gain an in-depth understanding of the basic characteristics of and RTW situation of cancer survivors to determine the factors that hinder their returning to work. This study can be used as a reference for employment services of the state, appropriate employment counseling and assisting unemployed cancer survivors in returning to work.

## Methods

The period investigated in this study was from 2004 to 2010, a total of 7 years, and the Labor Insurance Database was used as the main source of information. Labor insurance established by Taiwan government is a compulsory program for workers above 15 years and below 65 years of age intended to protect their rights and interests. First, we identified all employees covered by labor insurance using "Insured File" and "Enterprise File" in the Labor Insurance Database. All selected employee data included salary, company size, county and city where the company is located, changes in insurance, effective date of insurance, employment category and other related information. Combined with the Taiwan Cancer Registry, we identified employees with an initial diagnosis of cancer, thus establishing the cohort for this study. There were 136,342 eligible participants enrolled in this cohort study, including 69,619 patients returning to work and 66,723 patients not returning to work. In addition, we used the National Health Insurance Research Database to obtain inpatient and outpatient records, comorbidities, and cancer-related treatments (including surgery, radiation therapy, chemotherapy, and hormone therapy) as well as prediction of death for each case. This study was reviewed and approved by the Institutional Review Board of Tri-Service General Hospital.

### Covariables

Relevant variables were collected from the databases for the purpose of the study. Personal characteristics included age, gender, monthly salary (divided into ≤ 28,800, 28,800–38,200, and > 38,200 New Taiwan Dollars), medical care accessibility (divided into North, Middle, South, and East according to health insurance subdivision) and employment category. Health status included a medical history of major chronic diseases in the year prior to the cancer diagnosis (according to the International Classification of Diseases 9th edition, ICD-9). The clinical comorbidities included disorders of lipid metabolism (ICD-9-CM codes, 272), alcohol abuse (ICD-9-CM codes, 265.2, 291.1–291.3, 291.5–291.9, 303.0, 303.9, 305.0, 357.5, 425.5, 535.3, 571.0–571.3, 980.x, V11.3), cerebrovascular diseases (ICD-9-CM codes, 362.34, 430.x-438.x), chronic pulmonary diseases (ICD-9-CM codes, 416.8, 416.9, 490.x-505.x, 506.4, 508.1, 508.8), peptic ulcer diseases (ICD-9-CM codes, 531.x-534.x), renal diseases (ICD-9-CM codes, 403.01, 403.11, 403.91, 404.02, 404.03, 404.12, 404.13, 404.92, 404.93, 582.x, 583.0–583.7, 585.x, 586.x, V42.0, V45.1, V56.x), liver diseases (ICD-9-CM codes, 070.22, 070.23, 070.32, 070.33, 070.44, 070.54, 070.6, 070.9, 570.x, 571.x, 573.3, 573.4, 573.8, 573.9, V42.7), psychoses (ICD-9-CM codes, 293.8, 295.x, 296.04, 296.14, 296.44, 296.54, 297.x, 298.x), and depression (ICD-9-CM codes, 296.2, 296.3, 296.5, 300.4, 309.x, 311.xx).

Cancer diagnoses included cancer [according to the International Classification of Diseases for Oncology (ICD-O-3), oral cavity, 140–146, 148–149; major salivary glands, 142; nasopharyngeal, 147; esophagus, 150; stomach, 151; small intestine, 152; colon, rectum and anus, 153–154; liver and intrahepatic bile ducts, 155; gallbladder and extrahepatic bile ducts, 156; pancreas, 157; posterior peritoneal cavity and peritoneum, 158; unknown site of other digestive organs, 159 nasal, middle ear and paranasal sinuses, 160; larynx, 161; lung, bronchi and trachea, 162; thymus, heart and mediastinum, 163; bone, joint and articular cartilage, 170; connective tissue, subcutaneous tissue and other soft tissue, 171; Skin, 173; female breast, 174; uterus, 179; cervical, 180; uterus, 182; ovary, fallopian tube and broad ligament, 183; other female reproductive organs, 184; prostate; 185; testis, 186; Other male reproductive organs, 187; bladder, 188; kidney, renal pelvis and other urinary system structures, 189; eye and lacrimal gland, 190; brain, 191; other nervous system, 192; thyroid, 193; other endocrine glands, 194; leukemia, 196], initial diagnosis date, cancer type (tissue type and shape), cancer severity (differentiation stage and clinical tumor size), and treatment method (surgery, radiation therapy, and chemotherapy).

### Primary outcome

The main outcome of this study was RTW after an initial diagnosis of cancer. Full RTW was defined as the time in calendar days of sick leave until complete work resumption^13^. RTW was confirmed based on employment data from the Labor Insurance database. Unemployment was defined as the employee withdrawing from insurance and not being insured again for 5 years after the initial diagnosis of cancer. The secondary endpoint was the all-cause mortality after RTW within the follow-up period for workers with cancer.

### Statistical analysis

The SAS statistical software package (version 9.3, SAS Institute Inc., Cary, North Carolina) was used to analyze the descriptive statistics. Continuous variables are expressed as the mean and standard deviation, and categorical variables are expressed as frequencies and percentages. The independent samples t-test, chi-square test, Pearson product difference correlation, ANOVA and logistic regression were used for inferential statistics analyses. Univariate and multivariate adjustments for the Cox proportional hazard model were used to determine the risk factors that can significantly predict the presence or absence of returning to work. The fully adjusted model includes age, treatment, income range, industrial classification, company size, and cancer stage. The hazard ratio (HR) and 95% confidence interval (CI) of the HR are provided to indicate the degree of risk. Finally, the survival rates of survivors with different cancer stages were evaluated using Kaplan–Meier survival analysis. In this study, the statistical significance level α was defined as 0.05. A *p*-value lower than the significance level indicated statistical significance.

### Ethical approval

All procedures performed in studies involving human participants were in accordance with the ethical standards of the institutional and/or national research committee and with the 1964 Helsinki declaration and its later amendments or comparable ethical standards.

### Informed consent

Informed consent was obtained from all individual participants included in the study.

## Results

Table 1 provides a summary of returning to work at the fifth year for patients from a fixed cohort with an initial diagnosis of cancer. The average age reemployed patients was 47.6 ± 9.4 years old, and the majority were women (66.7%). The average age of those who did not return to work was 52.1 ± 9.7 years old, and male patients accounted for the majority (60.1%). In terms of personal disease factors, participants who returned to work had fewer comorbidities than those who did not return to work. In terms of disease treatment, the largest proportion of workers received operation (returning to work: 76.5%; non-returning to work: 47.7%) after cancer diagnosis. In terms of pathological grades, the majority of cancer survivors who returned to work had stage 2–4 disease. Female breast cancer accounted for the highest proportion (24.1%) of cancer survivors, followed by cervical (15.9%) and colorectal and anal (10.1%) cancer survivors.

**Table 1:** Demographic data of RTW group and non-RTW group in the 5th year.

Variable	ALL	RTW	nonRTW
	136,342	69,619 (51.1%)	66,723 (48.9^)
Age (year) mean ± SD	49.8 ± 9.8	47.6 ± 9.4	52.1 ± 9.7
Gender	63,247	23,169 (33.3%)	40,078 (60.1%)
**Comorbidity**			
Disorders of lipoid metabolism	11,997	5706 (8.2%)	6291 (9.4%)
Obesity	353	200 (0.3%)	153 (0.2%)
Alcohol abuse	2466	493 (0.7%)	1973 (2.9%)
Hypertension	24,741	10,566 (15.2%)	14,175 (21.2%)
Myocardial infarction	364	125 (0.2%)	239 (0.3%)
Congestive heart failure	1959	623 (0.9%)	1336 (2.0%)
Peripheral vascular disease	1070	440 (0.6%)	630 (0.9%)
Cerebrovascular disease	3261	1037 (1.5%)	2224 (3.3%)
Dementia	194	38 (0.1%)	156 (0.2%)
Chronic pulmonary disease	7276	2605 (3.7%)	4671 (7.0%)
Rheumatologic disease	1394	746 (1.1%)	648 (1.0%)
Peptic ulcer disease	15,514	5997 (8.6%)	9517 (14.3%)
Mild liver disease	19,189	6919 (10.0%)	12,270 (18.4%)
Hemiplegia or paraplegia	327	100 (0.1%)	227 (0.3%)
Renal disease	3117	1133 (1.6%)	1984 (2.9%)
Moderate or severe liver disease	1600	262 (0.4%)	1338 (2.0%)
Psychoses	667	253 (0.4%)	414 (0.6%)
Depression	3307	1597 (2.3%)	1710 (2.6%)
**Treatment**			
Operation	65,154	40,750 (76.5%)	24,404 (47.7%)
Radiotherapy	24,378	11,989 (22.6%)	12,389 (24.2%)
Chemotherapy	31,896	13,014 (24.6%)	18,882 (36.9%)
Hormone therapy	10,797	7333 (13.8%)	3464 (6.8%)
**Working district**			
Central	27,919	14,316 (20.6%)	13,603 (20.4%)
North	67,119	34,784 (50.0%)	32,335 (48.4%)
East	2788	1279 (1.8%)	1509 (2.3%)
South	38,222	19,085 (27.4%)	19,137 (28.7%)
Islands	294	155 (0.2%)	139 (0.2%)
**Income range**			
≤ 28,800	84,430	46,600 (66.9%)	37,830 (56.7%)
> 28,000–38,200	21,308	11,703 (16.8%)	9605 (14.4%)
> 38,200	30,604	11,316 (16.3%)	19,288 (28.9%)
**Industrial classification**			
Agriculture, Forestry, Fishing and Husbandry	9566	4760 (6.8%)	4806 (7.2%)
Mining and Quarrying	98	37 (0.1%)	61 (0.1%)
Manufacturing	42,511	21,936 (31.5%)	20,575 (30.8%)
Electricity and Gas Supply	451	146 (0.2%)	305 (0.4%)
Water Supply and Remediation Activities	891	349 (0.5%)	542 (0.8%)
Construction	15,464	6972 (10.0%)	8492 (12.7%)
Wholesale and Retail Trade	16,656	8677 (12.4%)	7979 (11.9%)
Transportation and Storage	9362	4203 (6.0%)	5159 (7.7%)
Accommodation and Food Service Activities	5527	2965 (4.2%)	2562 (3.8%)
Information and Communication	2033	1050 (1.5%)	983 (1.5%)
Financial and Insurance Activities	3991	2299 (3.3%)	1692 (2.5%)
Real Estate Activities	1561	766 (1.1%)	795 (1.2%)
Professional, Scientific and Technical Activities	3275	1715 (2.5%)	1560 (2.3%)
Support Service Activities	3666	1825 (2.6%)	1841 (2.7%)
Public Administration and Defense	2730	1337 (1.9%)	1393 (2.1%)
Education	2116	1254 (1.8%)	862 (1.3%)
Human Health and Social Work Activities	3215	1941 (2.8%)	1274 (1.9%)
Amusement and Recreation Activities	1567	852 (1.2%)	715 (1.1%)
Other Service Activities	11,662	6535 (9.4%)	5127 (7.7%)
**Company size**			
Shut down	13,272	6300 (9.0%)	6972 (10.4%)
Small	9994	5096 (8.1%)	4898 (8.2%)
Small and medium	30,095	15,470 (24.4%)	14,625 (24.5%)
Large	82,981	42,753 (67.5%)	40,228 (67.3%)
**Pathological stage**			
0	9165	7505 (24.4%)	1660 (9.2%)
1	14,586	10,307 (33.5%)	4279 (23.8%)
2	11,418	7588 (24.7%)	3830 (21.3%)
3	8372	4101 (13.3%)	4271 (23.7%)
4	5209	1261 (4.1%)	3948 (22.0%)
**Cancer type**			
Oral cavity	12,222	5715 (8.2%)	6507 (9.7%)
Major salivary glands	485	333 (0.5%)	152 (0.2%)
Nasopharyngeal	4116	2388 (3.4%)	1728 (2.6%)
Esophagus	2989	377 (0.5%)	2612 (3.9%)
Stomach	4467	1530 (2.2%)	2937 (4.4%)
Small intestine	463	222 (0.3%)	241 (0.4%)
Colon	8118	3889 (5.6%)	4229 (6.3%)
Rectum and anus	6348	3147 (4.5%)	3201 (4.8%)
Liver and intrahepatic bile ducts	16,091	3733 (5.4%)	12,358 (18.5%)
Gallbladder and extrahepatic bile ducts	743	190 (0.3%)	553 (0.8%)
Pancreas	1579	132 (0.2%)	1447 (2.2%)
Posterior peritoneal cavity and peritoneum	252	105 (0.2%)	147 (0.2%)
Nasal, middle ear and paranasal sinuses	366	181 (0.3%)	185 (0.3%)
Larynx	820	417 (0.6%)	403 (0.6%)
Lung, bronchi and trachea r	9432	1525 (2.2%)	7907 (11.8%)
Thymus, heart and mediastinum	664	310 (0.4%)	354 (0.5%)
Bone, joint and articular cartilage	274	151 (0.2%)	123 (0.2%)
Connective tissue and other soft tissue	830	440 (0.6%)	390 (0.6%)
Skin	2935	2018 (2.9%)	917 (1.4%)
Female breast	23,292	16,768 (24.1%)	6524 (9.8%)
Cervix	13,708	11,048 (15.9%)	2660 (4.0%)
Uterus	3174	2282 (3.3%)	892 (1.3%)
Ovary, fallopian tube and broad ligament	2489	1380 (1.9%)	1109 (1.7%)
Prostate	1647	802 (1.1%)	845 (1.3%)
Testis	398	308 (0.4%)	90 (0.1%)
Bladder cancer	2528	1476 (2.1%)	1052 (1.6%)
Kidney	1532	857 (1.2%)	675 (1.0%)
Renal pelvis and other urinary structures	1245	607 (0.9%)	638 (0.9%)
Eye and lacrimal gland	144	90 (0.1%)	54 (0.1%)
Brain	1169	364 (0.5%)	805 (1.2%)
Thyroid	5439	4295 (6.2%)	1144 (1.7%)
Leukemia	4618	1977 (2.8%)	2641 (3.9%)
Others	605	337 (0.5%)	268 (0.4%)

In Supplementary Fig. 1, the OR of RTW rapidly increased after the 2nd year in all cancer survivors. A rapid decline was noted after the 4th year. This implied that cancer survivors might return to work within the first 2–6 years after diagnosis with cancer.

Table 2 shows the number of deaths, survivors, employed individuals, and the number of people who left the workplace in the 1st to 5th years after the initial diagnosis of cancer and the 10 most common cancers. A total of 70.4% of cancer survivors remained employed through the first year after the initial diagnosis of cancer, accounting for 83.4% of the cancer survivors. After the fifth year, 51.1% of the cancer survivors remained employed, accounting for 78.7% of the cancer survivors. In the 1st year after the initial diagnosis of cancer, patients with cervical cancer (86.1%), female breast cancer (83.6%) and thyroid cancer (82.6%) represented the highest proportion of cancer survivors who remained employed; in the 5th year, patients with cervical cancer (80.6%), thyroid cancer (79.0%), and female breast cancer (72.0%) represented the highest proportion among those who remained employed.

**Table 2:** Longitudinal distribution of workers with cancer in 5 years (2004–2010).

Cancer type	Work status	Time				
		1st year	2nd year	3rd year	4th year	5th year
All cancers (N = 136,342)	Death	21,616	32,974	39,764	44,389	47,851
	Change work	89,658	75,076	66,917	61,695	58,125
	RTW	6312	9238	10,472	11,075	11,494
	Unemployment	18,756	19,054	19,189	19,183	18,872
	Survival rate (%)	84.15	75.82	70.84	67.44	64.90
	Employment rate (%)	70.39	61.84	56.76	53.37	51.06
Breast (N = 23,292)	Death	353	923	1552	2114	2621
	Change work	18,193	16,652	15,509	14,700	14,107
	RTW	1279	2024	2394	2548	2661
	Unemployment	3467	3693	3837	3930	3903
	Survival rate (%)	98.5	96.0	93.3	90.9	88.7
	Employment rate (%)	83.6	80.2	76.9	74.1	72.0
Liver and intrahepatic bile ducts (N = 16,091)	Death	6535	8479	9675	10,488	11,080
	Change work	7573	5605	4498	3778	3281
	RTW	414	477	462	456	452
	Unemployment	1569	1530	1456	1369	1278
	Survival rate (%)	59.4	47.3	39.9	34.8	31.1
	Employment rate (%)	49.6	37.8	30.8	26.3	23.2
Cervix (N = 13,708)	Death	212	470	674	814	895
	Change work	10,866	10,044	9563	9274	9091
	RTW	940	1435	1701	1842	1957
	Unemployment	1690	1759	1770	1778	1765
	Survival rate (%)	98.5	96.6	95.1	94.1	93.5
	Employment rate (%)	86.1	83.7	82.2	81.1	80.6
Oral cavity (N = 12,222)	Death	1880	3435	4101	4596	4992
	Change work	8253	6541	5776	5231	4844
	RTW	540	749	820	851	871
	Unemployment	1549	1497	1525	1544	1515
	Survival rate (%)	84.6	71.9	66.4	62.4	59.2
	Employment rate (%)	71.9	59.6	54.0	49.8	46.8
Trachea, bronchus, and lung (N = 9432)	Death	3353	5181	6285	6871	7219
	Change work	4719	2960	2027	1555	1295
	RTW	246	292	265	238	230
	Unemployment	1114	999	855	768	688
	Survival rate (%)	64.5	45.1	33.4	27.2	23.5
	Employment rate (%)	52.6	34.5	24.3	19.0	16.2
Colon (N = 8118)	Death	941	1673	2182	2508	2754
	Change work	5548	4551	3944	3571	3315
	RTW	319	472	521	546	574
	Unemployment	1310	1422	1471	1493	1475
	Survival rate (%)	88.4	79.4	73.1	69.1	66.1
	Employment rate (%)	72.3	61.9	55.0	50.7	47.9
Rectum and anus (N = 6348)	Death	480	1011	1432	1740	1977
	Change work	4516	3744	3264	2917	2692
	RTW	266	413	442	451	455
	Unemployment	1086	1180	1210	1240	1224
	Survival rate (%)	92.4	84.1	77.4	72.6	68.9
	Employment rate (%)	75.3	65.5	58.4	53.1	49.6
Thyroid gland (N = 5439)	Death	68	94	113	130	153
	Change work	4086	3816	3647	3539	3446
	RTW	405	599	719	800	849
	Unemployment	880	930	960	970	991
	Survival rate (%)	98.7	98.3	97.9	97.6	97.2
	Employment rate (%)	82.6	81.2	80.3	79.8	79.0
Leukemia (N = 4618)	Death	1057	1543	1761	1904	2013
	Change work	2638	2059	1815	1654	1545
	RTW	212	330	380	420	432
	Unemployment	711	686	662	640	628
	Survival rate (%)	77.1	66.6	61.9	58.8	56.4
	Employment rate (%)	61.7	51.7	47.5	44.9	42.8
Stomach (N = 4467)	Death	1253	1850	2136	2296	2404
	Change work	2489	1857	1567	1393	1289
	RTW	154	206	227	236	241
	Unemployment	571	554	537	542	533
	Survival rate (%)	71.9	58.6	52.2	48.6	46.2
	Employment rate (%)	59.2	46.2	40.2	36.5	34.3

**Figure 1:**
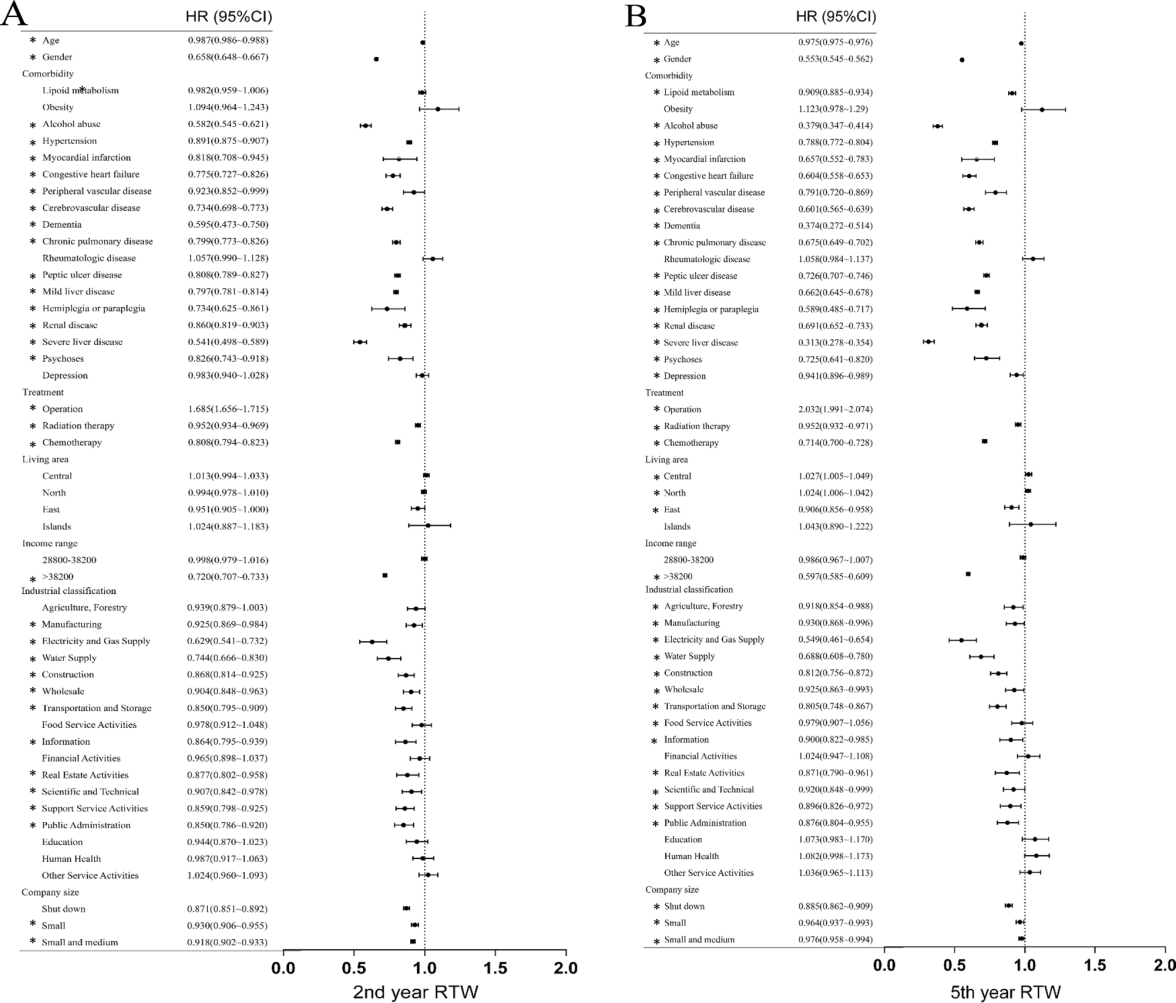
Univariate association between independent variables and RTW in the 2nd and 5th year.

**Figure 2:**
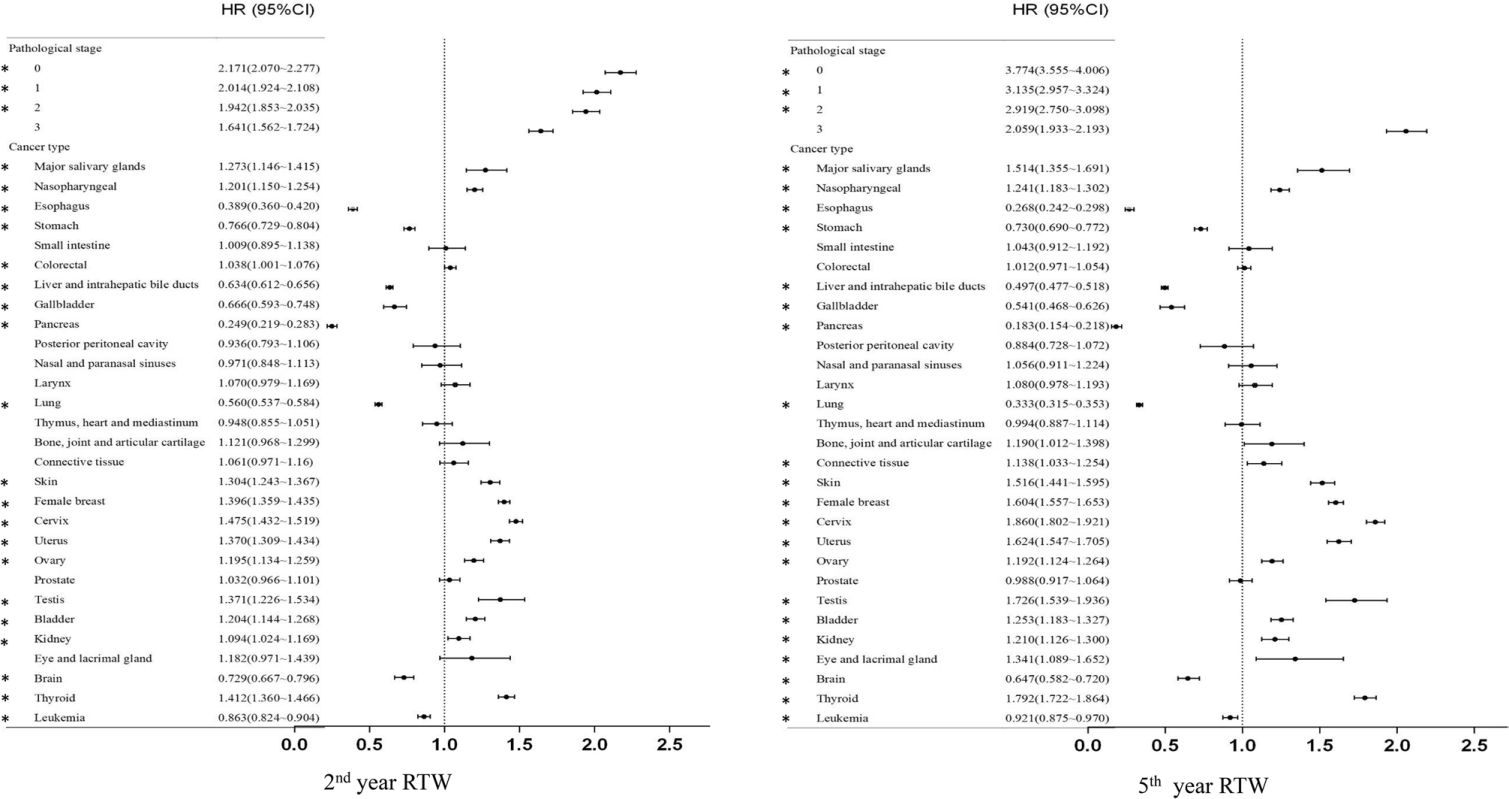
Univariate association between different cancer types and RTW in the 2nd and 5th year.

Figures 1 and 2 show the results of the univariate analysis of factors that affect returning to work in the 2nd and 5th years after the initial diagnosis. The results showed that age (older) and gender (male) were negatively associated with returning to work. Alcohol abuse, hypertension, myocardial infarction, heart failure, peripheral arterial disease, cerebrovascular disease, dementia, chronic pulmonary diseases, peptic ulcer, mild liver disease, hemiparesis, kidney disease, moderate-severe liver diseases and mental illness were significantly negatively associated with RTW in the 2nd and 5th years, and dyslipidemia and depression were significantly negatively associated with returning to work only in the fifth year. Surgical treatment showed a significant positive relationship with RTW in the 2nd and 5th years. Radiation therapy and chemotherapy were significantly negatively associated with returning to work in the 2nd and 5th year. Based on histopathological staging, compared to the highest stage (stage 4), lower stages were significantly positively associated with returning to work in the 2nd and 5th year. In terms of cancer types, we used oral cancer, which is prevalent in Taiwan and Southeast Asia, as the reference. The results showed that patients with salivary gland cancer, nasopharyngeal carcinoma, osteocarcinoma, chondrocarcinoma, sarcoma, skin cancer, female breast cancer, female genital cancer, testicular cancer, bladder cancer, renal cancer and thyroid cancer were significantly associated with increased rates of returning to work in the 5th years. In contrast, esophageal cancer, gastric cancer, hepatic cancer and intrahepatic cholangiocarcinoma, gallbladder cancer and extrahepatic cholangiocarcinoma, pancreatic cancer, thoracic cancer, brain cancer, and leukemia were significantly associated with reduced returning to work in the 5th year.

**Figure 3:**
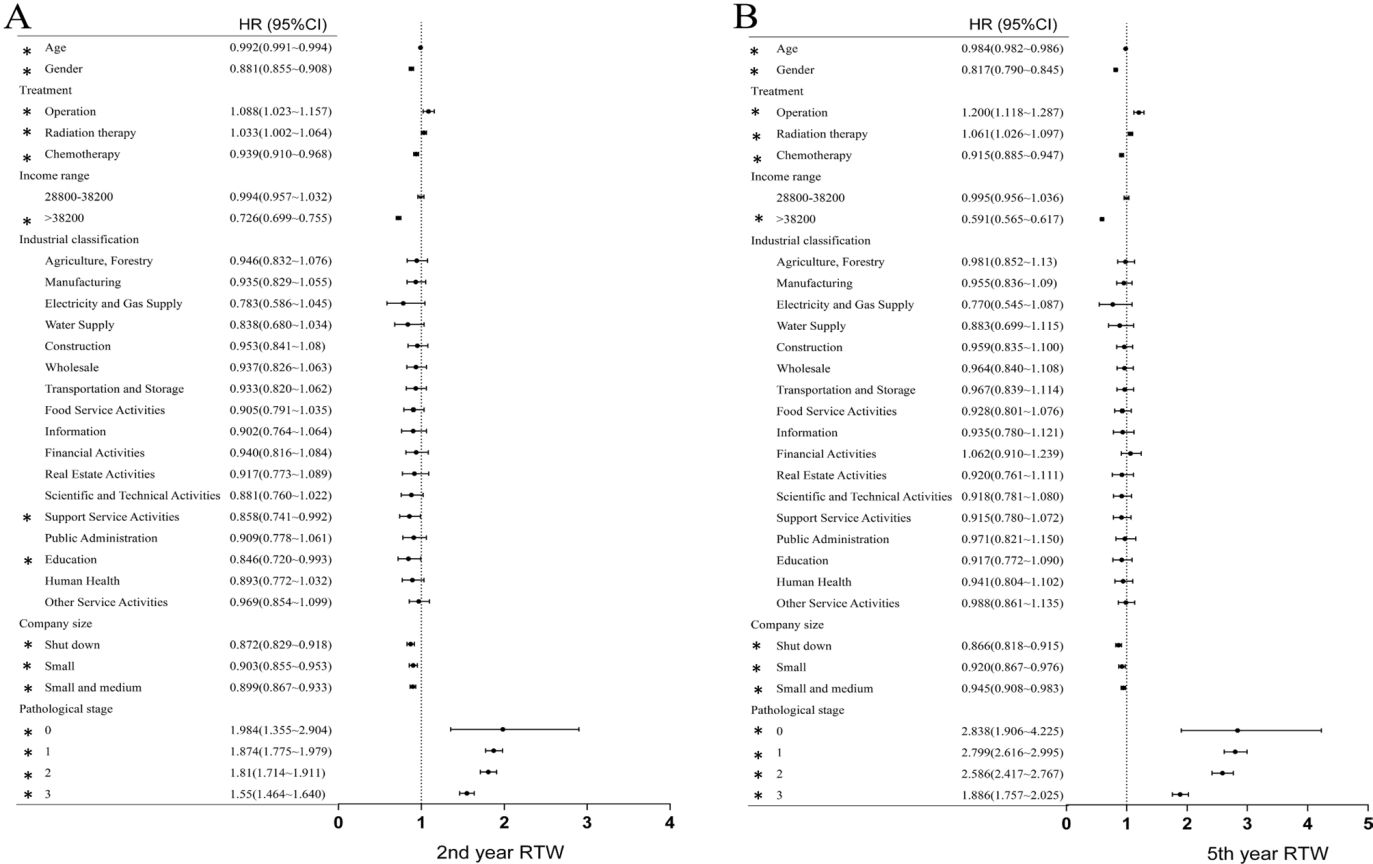
Multivariate association between independent variables and RTW in the 2nd and 5th year.

Figure 3 presents the relationship between the different variables and the occurrence of returning to work in the 2nd and 5th years. The results showed that the HR of returning to work in the 2nd and 5th years was decreased for patients with the following characteristics: older age, male sex, difference in salary level greater than 38,200 New Taiwan Dollars, receiving chemotherapy, working in medium/small scale industry, and advanced cancer stage. In contrast, for patients who underwent surgery or radiotherapy, the HR of returning to work in the 2nd and 5th years was still increased. The most notable change was for radiation therapy. In the univariate analysis, radiation therapy was a negative factor for returning to work, but it was a positive factor in the multivariate analysis.

**Figure 4:**
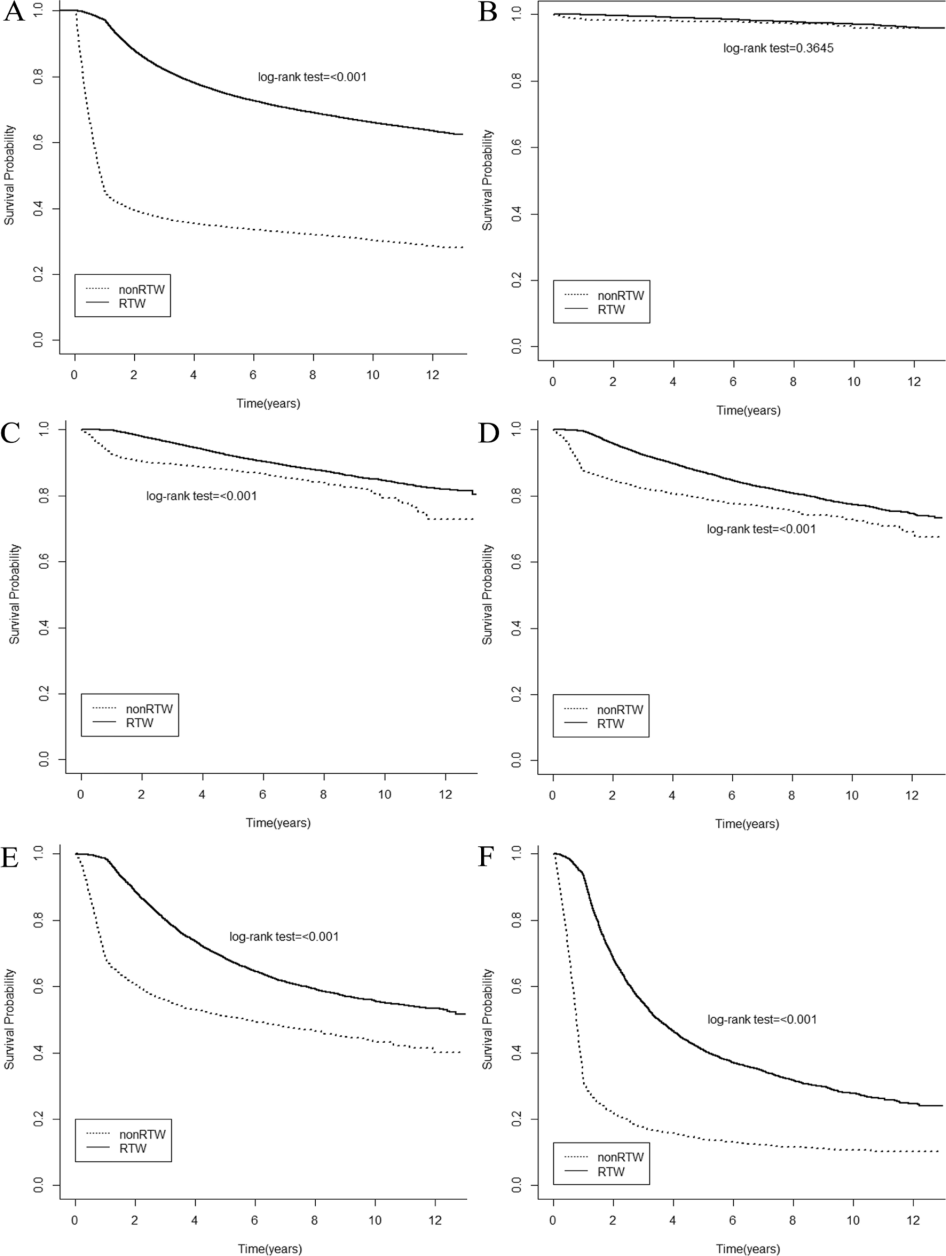
Kaplan–Meier curve for all-cause mortality categorized by all cancer stages. (**A**) all stages (**B**) stage 0; (**C**) stage 1; (**D**) stage 2; (**E**) stage 3; (**F**) stage 4.

Figure 4 displays the survival rates of survivors with different cancer stages (A: all stages; B: stage 0; C: stage 1; D: stage 2; E: stage 3; F: stage 4) evaluated using Kaplan–Meier survival analysis. The survival rates of all cancer stages were significantly higher in the returning to work group than in the non- returning to work group (*p* < 0.001). The returning to work group had significantly higher survival rates than the non- returning to work group for survivors with stage 1, 2, 3, and 4 diseases (*p* < 0.001). Table 3 shows the association between returning to work and all-cause mortality. Patents who returned to work had significantly reduced risk of all-cause mortality with an HR of 0.46 (95%CI: 0.44–0.48, *p* < 0.001) after fully adjusting for the variables.

**Table 3:** Associations between returning to work and all-cause mortality.

	Unadjusted HR (95% CI)	*p* value	Fully adjusted HR (95% CI)	*p* value
RTW	0.25 (0.25–0.26)	< 0.001	0.46 (0.44–0.48)	< 0.001

Adjusted covariates: age, treatment, income range, industrial classification, company size, cancer stage, return to work.

## Discussion

The aim of this study was to analyze the return to work of cancer survivors in Taiwan by combining Taiwan’s Labor Insurance Database, National Health Insurance Research Database and Taiwan Cancer Registry. This study used a retrospective cohort study design to establish a cohort of cancer survivors with an initial diagnosis of cancer from 2004 to 2010. From the beginning to the end of the observation, there were no newly added research cases, and no study cases remained. This study found that among the patients with an initial diagnosis, 70.4% of the workers remained employed through the first year, accounting for 83.4% of the cancer survivors. In the fifth year after the diagnosis, 51.1% of the patients remained employed, accounting for 78.7% of cancer survivors, a decrease from the first year. The results of our study showed that the important factors affecting whether patients with an initial diagnosis of cancer returned to work include age, gender, salary level, treatment method, enterprise size and cancer stage and whether returning to work was the key factor affecting the future survival of patients.

The relationship between cancer and returning to work has been presented in numerous studies. Fantoni et al. demonstrated that a high proportion of employed patients with breast cancer returned to work in 36 months after treatment^14^. In a recent study, the risk factors affecting returning to work of cancer survivors included personal, employment, and socioeconomic factors^15^. Chen et al. demonstrated that RTW may have a beneficial effect on the survival of patients with oral cancer in Taiwan^16^. Among the cancer and symptom factors**,** in our study, the chances of returning to work were higher for the patients who received surgical treatment. However, if patients received chemotherapy and radiotherapy, the chances of returning to work were relatively low. The reasons for this may be that the patients who received only surgical treatment had their cancer detected at an early stage. Patients with relatively advanced stage disease may require chemotherapy and radiotherapy. In addition, chemotherapy and radiotherapy require 3–6 months, and patients need to visit the hospital or outpatient clinic for several courses of treatment and are more prone to complications or discomfort. Consistent with our findings, a study on breast cancer survivors with a median monthly follow-up of 36 months found that chemotherapy or radiotherapy restricted or postponed returning to work, possibly due to end-stage cancer patients requiring multiple treatment strategies^14^. The multifactor analysis indicated that changes in receiving radiotherapy were a positive factor for returning to work; however, the possible reason for this result was that our study did not group survivors according to multiple treatment strategies. For cancer staging, compared to patients with stage 4 cancer, patients with lower-stage cancer (e.g., stage 1) had a greater chance of returning to work. Studies from other counties also showed that patients with terminal cancer or palliative treatments were associated with a lower return to work rate^17,18^. In terms of cancer types, previous studies have shown that liver cancer, pulmonary cancer, brain cancer, blood cancer, gastrointestinal cancer, pancreatic cancer, head and neck cancer, and gynecological cancer are all significantly correlated with unemployment or losing jobs^18–22^. Another study found that male and female genital cancer, skin cancer and breast cancer had the highest return to work rate 2 years after a cancer diagnosis^23^. Cervical cancer and female breast cancer survivors had a higher return to work rate, possibly due to the continued promotion of Pap smear screening and breast cancer screening by the Taiwan Health Promotion Administration^24^. Women over the age of 30 can receive an annual free Pap smear examination and women aged 45–69 years old or 40–44 years old with a family history of breast cancer in first- or second-degree relatives can receive free mammography once every 2 years. These screening programs can greatly improve the diagnostic rate of early cervical cancer and breast cancer, thereby reducing disability and work-related issues in working women^25,26^.

For demographic factors, based on age, the older an individual is, the lower the chance of the patient returning to work, which may also be related to the retirement age in Taiwan (according to the Ministry of Labor’s statistics, Taiwan's actual retirement age from 2010 to 2015 was 63.3 years for men and 60.6 years for women. In this study, the average age of patients in the fifth year after the initial diagnosis was 49.8 years old, the average age of patients who returned to work was 47.6 years old, and the average age of patients who did not return to work was 52.1 years old). Because the age at diagnosis was close to the retirement age, patient motivation to return to work may be decreased. In a Danish study, compared with younger patients, older patients (50–60 years) had a higher unemployment rate^27^. In addition, studies have shown that demographic factors that affect return to work included gender and low socioeconomic levels^18^. Marino’s study addressing gender and return to work showed that among patients who were still alive 2 years after a cancer diagnosis, older men returned to work later than did older women, but married men returned to work earlier than did married women^28^. In our cohort, women were more likely to return to work than men, probably due to the high proportion of women with cervical cancer and breast cancer, which are mainly identified at early stages. Oral cancer is more common in Taiwanese men and is mostly caused by chewing betel nuts and smoking. In the fifth year, returning to work of men with oral cancer was only half that of women with cervical cancer and breast cancer.

Survival prognosis is an important indicator for cancer survivors. After confirming prognostic factors that affect survival, health education, rehabilitation and treatment are performed to improve the survival and the quality of life of patients. In our study, returning to work significantly affected the prognosis of patients, especially for patients with stage 4 cancer. Daily physical performance, for example, can be measured with the US Eastern Cooperative Oncology Group and Karnofsky performance scales In a study of pancreatic cancer, poor daily physical performance was significantly correlated with a poor prognosis^29^. In patients with breast cancer that metastasized to the brain, physical performance in the presence of metastasis can predict survival^30^. Good physical performance is especially important for patients with advanced stage cancer, indicating that return to work can effectively predict the survival of these patients^31^.

An advantage of this study is the analysis of big data from a labor insurance and health insurance database in Taiwan. At the end of 2016, there were a total of 10,165,434 people were enrolled in labor insurance. The 2 databases were merged, and information related to the diagnoses and treatment of cancer survivors and changes in employment over 11 years were tracked. A limitation of this study is that the database did not include other important factors for determining the return to work of cancer survivors, such as education level, family support, personal physical performance status, work effort level, etc. As a result, there are limitations related to the inferences that can be made.

In this study, important factors for returning to work RTW included chronic diseases, gender, age, salary, and cancer stage. In particular, we also found that returning to work was significantly associated with the survival of the patients. For the country and society, the return to work of patients with cancer can boost the social labor force and promote the national economy. For working-age patients, a cancer diagnosis and subsequent return to work after treatment is an important transition milestone from being a cancer patient to being a cancer survivor. Returning to work indicates recovery. The path to the return to work of cancer survivors requires not only interdisciplinary professional intervention but also the assistance of the state, society, and employers. Strengthening existing returning to work policies and assisting high-risk populations to return to work after treatment should be a priority to protect these individuals’ right to work and to maintain the overall labor force.

## Supplementary Information

Supplementary Information 1.
