# Supplementary figures and images for: Life expectancy estimations and determinants of return to work among cancer survivors over a 7-year period
Source: Sci Rep. 2021 Jun 18;11:12858. doi: 10.1038/s41598-021-92306-9 (PMC8213801; doi:10.1038/s41598-021-92306-9)

**Supplementary Figure 1.** The OR of RTW in all cancer survivors

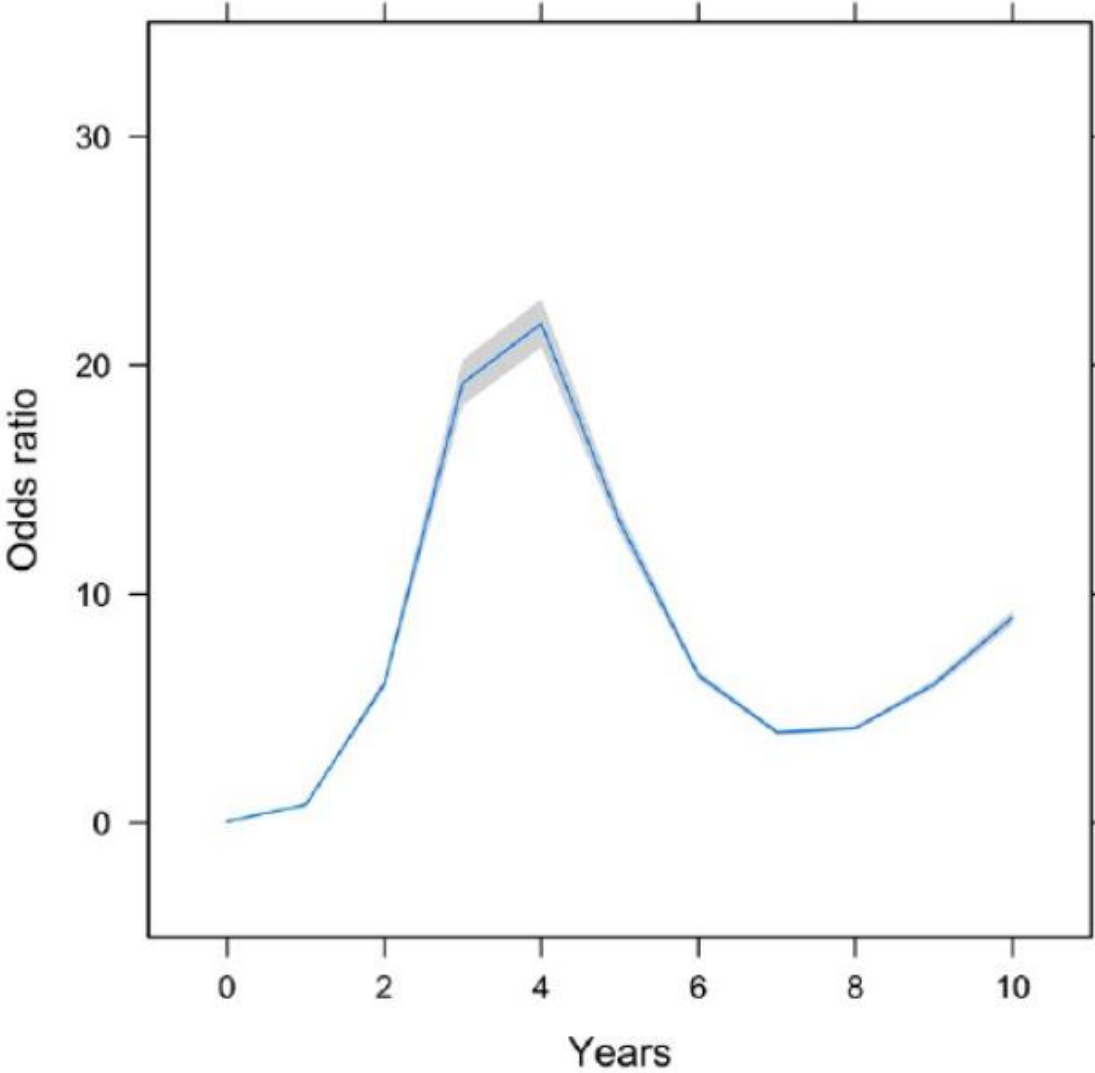

Supplement: Supplementary file 1 — Supplementary Information 1. [file 41598_2021_92306_MOESM1_ESM.pdf]
